# Sustained self-regulation of energy intake. Loss of weight in overweight subjects. Maintenance of weight in normal-weight subjects

**DOI:** 10.1186/1743-7075-7-4

**Published:** 2010-01-19

**Authors:** Mario Ciampolini, David Lovell-Smith, Massimiliano Sifone

**Affiliations:** 1 Unit of Preventive Gastroenterology, Department of Paediatrics, Università di Firenze, 50132 Florence, Italy; 2 Department of General Practice and Primary Health Care, University of Auckland, Auckland, New Zealand; 3 Department of Statistics, Università di Firenze, Florence, Italy

## Abstract

**Background:**

Dietary restraint is largely unsuccessful for controlling obesity. As an alternative, subjects can easily be trained to reliably recognize sensations of initial hunger (IH) a set of physiological sensations which emerge spontaneously, not necessarily at planned mealtimes, and may be the afferent arm of a homeostatic system of food intake regulation. Previously we have reported that IH is associated with blood glucose concentration (BG) below 81.8 mg/dL (4.55 mmol/l), (low blood glucose, LBG), and that a pattern of meals in which IH is present pre-meal (IHMP) improved insulin sensitivity, HbA1c and other cardiovascular risk factors. Here we report the effect upon weight in overweight and normal weight subjects.

**Objective:**

To investigate whether the IHMP is associated with sustained loss of weight in overweight subjects over a 5 month period.

**Methods:**

Seventy four overweight subjects (OW: BMI > 25) and 107 normal weight (NW) subjects were randomly allocated to either trained (OW: N = 51; NW N = 79) or control (OW: N = 23; NW: N = 28) groups. All subjects were allocated post-randomization into either low or high mean pre-meal BG groups (LBG and HBG groups) using a demarcation point of 81.8 mg/dL.

**Results:**

A significant longitudinal decrease was found in body weight (trained NW: -2.5 ± 4.6 kg; OW -6.7 ± 4.5 kg; controls: NW +3.5 ± 4.0 kg and OW -3.4 ± 4.0 kg; P = 0.006 and 0.029) and in energy intake, mean BG, standard deviation of diary BG (BG as recorded by subjects' 7-day diary), BMI, and arm and leg skin-fold thickness in (OW and NW) HBG subjects. OW LBG subjects significantly decreased body weight (trained: -4.0 ± 2.4 kg; controls: -0.4 ± 3.7 kg; P = 0.037). 26 NW LBG subjects showed no longitudinal difference after training as did 9 control subjects.

**Conclusion:**

Over a 5 month period the IHMP resulted in significant loss of weight in OW subjects compared to controls practicing dietary restraint. NW subjects maintained weight overall, however NW HBG subjects also lost weight compared to controls.

## Background

The adverse effects of obesity are well known and include cardiovascular disease, type 2 diabetes and hypertension as well as gall bladder disease, osteoarthritis, endocrine disorders, sleep apnoea, social exclusion and depression [1,2]. More than 1.1 billion adults worldwide are overweight, and 312 million of them are obese [2]. Ten per cent of school-aged children world-wide are estimated to be overweight, and of these, one quarter is obese [3]. With a prevalence of overweight children at over 35% and obese children over 13% (2003-2004 figures) the United States population is among the most obese in the world [4]. The prevalence of overweight children is lower in developing countries but is rising [5].

In adults, the decision to eat depends upon conditioned responses to external cues such as set mealtimes, others eating and highly palatable, available food (conditioned eating) as well as on physiological bodily sensations that reflect changes in the concentration of blood nutrients, including blood glucose [6] (unconditioned eating). Because conditioned eating tends to predominate, the feasibility of self-regulation of energy intake in an obesogenic environment has been questioned [7-9]. Dietary regimes that attempt to restrain eating have been largely unsuccessful [1,2].

The homeostatic systems that ensure constancy in osmotic pressure and body temperature rely for afferent information on bodily sensations (thirst and the sensation of heat respectively) [10]. We have trained subjects to reliably recognize comparable eating-related sensations that we group under the term *initial hunger *(IH) [11]. The necessity for such interoceptive information in homeostatic regulation has been recognized elsewhere [12-15]. We train subjects to adjust their meal-by-meal energy intake to ensure the pre-meal attainment of IH and its associated low BG concentration, three times per day. We term this routine the *Initial Hunger Meal Pattern *(IHMP) [16]. We suggest that IH is not conditioned by mealtime or other external cue and that the IHMP thus represents unconditioned eating. This contention is supported by our observation that in the early days of training subjects find that IH arises unexpectedly, often occurring at times far from usual mealtimes.

Elsewhere we found the IHMP was significantly associated with mean BG lower than 81.8 mg/dL (LBG) in weekly self-report diaries, and with improved insulin sensitivity, HbA1c and other cardiovascular risk factors in mixed body weight groups (NW and OW). Moreover, not only OW but NW HBG subjects lost weight under the IHMP supporting the notion that HBG and insulin resistance are important in the development of overweight [16]. Since BG concentration is a reliable index of energy availability to body cells [6], we hypothesized that the IHMP might allow for meal by meal homeostatic energy balance and weight regulation, and might be more effective than dietary methods that rely on restraint based on weekly or monthly measurements of weight. We now report the effect of the IHMP upon weight in OW and NW subjects.

## Methods

### Eligibility criteria

**Figure 1:**
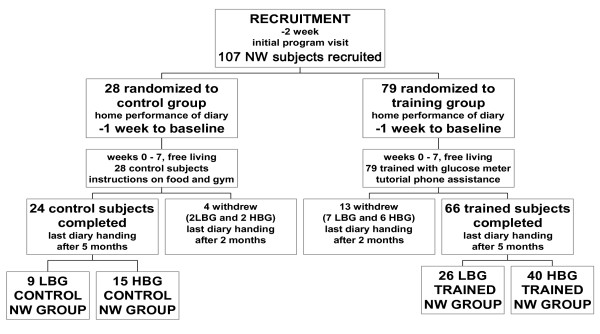
**Consort flow chart of NW subjects and investigation design**. Randomized and controlled 5-month clinical investigation (and drop outs) to study the effect of IHMP on normal body weight after training.

**Figure 2:**
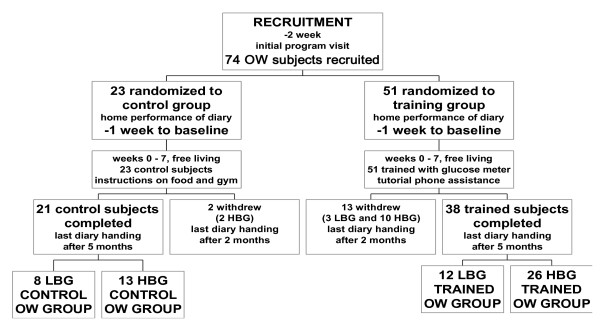
**Consort flow chart of OW subjects and investigation design**. Randomized and controlled 5-month clinical investigation (and drop outs) to study the effect of IHMP on body weight over 25 BMI after training.

A total of 181 subjects were recruited by the Paediatric Gastroenterology Unit of Florence University between 1995 and 2000 into two separate lists (Figures 1 and 2). Subjects showed no morphological, physical or biochemical signs of organic disease [17,18]. Subjects with impaired glucose tolerance (fasting plasma-glucose > 115 mg/dL (6.4 mmol/l)), as well as subjects suffering from non-insulin dependent diabetes mellitus (NIDDM), celiac, inflammatory bowel, liver, heart, brain and kidney diseases were excluded from recruitment. Informed consent was obtained from all participants. The local Hospital Ethics Committee approved the study in compliance with the Helsinki Declaration.

### The intervention

Subjects were trained in the IHMP, first by identifying IH, which was guided by consistency in subjective sensations and the association of these sensations with BG measurement.

### Identification of IH [11]

The explorative search for a subject's own signalling system took place during two instruction visits and a variable number of phone calls over the following seven weeks. Subjects were asked to ignore meal times at first, and to attend only to their sensations of hunger. At the earliest spontaneous arousal of sensations of hunger (IH) subjects were instructed to take note of the identified sensation, measure glucose concentrations with a portable instrument and consume a meal [11]. Subjects reported IH as gastric pangs, sensations of emptiness and hollowness and mental or physical weakness [11]. In the first three training days, before the IHMP was established, IH typically arose spontaneously and unexpectedly during usual activity often far from usual meal times (up to 48 hours, mean 2 hours) supporting the idea that IH is physiological and is not conditioned by external stimuli.

### Training in the IHMP

IH was cultivated pre-meal by adjusting composition, portion size or timing of food intake. After a few days of trial and error, and sometimes irregular mealtimes, subjects were able to arrange their food intake so that IH appeared before the usual three mealtimes per day with an average error of half-an-hour in 80% of instances [19]. If they overate at a given meal, subjects received feedback within a few hours since initial hunger did not appear pre-meal at the subsequent mealtime. This immediate feedback allowed for compensation by delaying, skipping or reducing the subsequent meal(s) (portion size and composition) to ensure a return of pre-mealtime initial hunger three times a day. Subjects were instructed to start a meal within 1 hour of the appearance of IH, and were prohibited from sustaining hunger for longer than 1 hour, to avoid BG declines below 60 mg/dL. Telephone assistance was provided so that subjects could describe their hunger sensations and times of occurrence, and to report pre-prandial BG and meal composition. With respect to content, up to 1 kg of fruit/vegetables per day was recommended [19]. Physical exercise for half an hour a day was also encouraged although we were unable to document any change in either physical exercise or time spent in bed. Energy intake decrease (via smaller portion size and slightly lower numbers of meals per day) and increase in vegetable intake were therefore the significant variables in achieving initial hunger as in other investigations [11,19-23], and were negatively correlated (see results). The generally consistent association between IH and low BG measurement gave confidence in the reliability of the sensations of IH although we found that BG measurements taken less than 1 hour after taking even a few grams of food, after changes in ambient temperature, after physical activity such as walking or cycling and when under psychic stress were misleading since they did not correlate well with IH. Subjects repeated and refined this procedure three times a day for at least two weeks, and became able to accurately estimate pre-meal BG by their experience of IH [11]. Training ended after the first 7 weeks to be resumed only at investigation end. Thus after the first 7 weeks, subjects relied upon the identified subjective sensation (IH) alone, as the signal to begin a meal.

Control and training subjects were visited at baseline, after the 7 weeks of training, and at investigation end 5 months from baseline. The visits included clinical assessment, measurement of weight and BMI, diary handing, suggestions on compliance where appropriate, and validation of BG measurements [19-23]. Each subject measured his or her own blood sample by portable glucometer calibrated against the hospital laboratory autoanalyzer. Seven-day home diaries reported BG measurements before the three main meal times. Subjects were also instructed to perform half an hour per day of physical exercise, and consume up to 1 kg fruit and vegetables. This purpose of the vegetables was to prevent distress from excessive hunger when IH appeared half an hour or more before mealtimes.

### Control groups

Control subjects were given information on food energy content and on recommended vegetable intake and physical activity similar to the trained subjects. The control OW subjects were encouraged to lose weight.

### Study objective

We wished to investigate whether the IHMP is associated with loss of weight over a 5 month period.

### Outcomes

#### Primary endpoint

The primary endpoint was weight (expressed as BMI) at 5 months from baseline compared to controls.

#### Secondary endpoints

At investigation end the following additional variables were assessed:

1. Pre-meal BG and BG standard deviation (BG SD)

2. Arm and leg skinfold thickness [13,19].

3. Fruit and vegetable intake.

4. Systolic and diastolic blood pressures.

The seven-day home diaries also recorded food intake, bedtime hours and outdoor and gym hours [19-23].

### Sample size

Preliminary work in similar patients found BMI in the intervention group to be 26.6 (SD 3.6) mg/dL; and in the control group 29.0 (SD 3.5) mg/dL [19]. Based on these figures, our sample size calculations suggested that we need a minimum of 21 subjects in each comparison group to detect a difference of 2.0 in group means, with a power of 80.% and a 1 sided alpha of 0.05.

### Randomization

A dietician assigned subjects to either NW or OW lists according to body mass index (BMI) either lower or higher than 25.0. Subjects were allocated into trained and control groups in blocks (3:1) randomised by random numbers (Figure 1 and 2) [24].

### Statistical methods

Values are expressed as means ± SD. Yates test and two-tailed Student's *t*-test on paired or unpaired samples with different variances according to data requirements were used to analyze the statistical significance of differences and correlations. The significance was set at P < 0.05 for single measurements. The Bonferroni correction was applied when required in the evaluation of results from multiple comparisons The Chi-square for trend assessed the global significance of improvements in these trials. In multiple analyses between the same groups, the "<" symbol indicates the analysis of least significant P. MANOVA was performed on multiple variables to assess the training effect and main factors in training [24]. Evaluation of model assumptions was always checked.

Custom-made software was used to tabulate data for statistical analyses. Microsoft Excel (Microsoft Corp., USA) and SAS 8 (SAS Institute Inc., Cary, NC, USA) were used for data presentation and statistical analyses.

## Results

### Flow of participants

Figures 1 and 2 show the flow of participants through each phase of the study.

### Baseline demographics

Baseline (*i.e*. before training) values of mean age, school education years, body weight, height, BMI, skinfold thickness, arm and leg circumferences, systolic and diastolic blood pressure did not significantly differ between the trained and the control NW and OW groups (in subgroups also, see section on post-hoc analysis below). The lowest P value on baseline differences between control and trained groups was in leg quadriceps thickness for NW HBG comparison (P = 0.07; Tables 1, 2, 3).

**Table 1:** Over-weight groups at baseline and at investigation end: composition, compliance and effects of training (IHMP) on diary reports and anthropometry.

	Control		Trained	
	**Baseline**	**After 5 mo**.	**Baseline**	**After 5 mo**.

Number of subjects and gender	13F + 8M		23F + 15M	

Schooling^1^	11.1 ± 2.7		12.0 ± 3.0	

Age ^1^	33.7 ± 14.4		36.7 ± 12.6	

Subjects showing BG decrease ^2^	3/21 (14.3%)		24/38 (63.2%) ***,^*a*^	

BG group mean pre-meal^3^	85.7 ± 9.0	89.3 ± 8.2 *,^*b*^	86.8 ± 8.7	78.8 ± 6.8 ***,^a^***,^*b*^

Subjects < 81.8 mg/dL^4^	8 (38.1%)	5 (33.8%)	12 (31.6%)	29 (76.3%) ***,^a^***,^*b*^

Vegetable intake^5^	246 ± 188	427 ± 263 **,^*b*^	274 ± 166	449 ± 218 ***,^*b*^

Fruit intake^5^	193 ± 155	173 ± 160	221 ± 122	266 ± 174

Energy intake^1^	1728 ± 551	1310 ± 532 **,^*b*^	1756 ± 585	1069 ± 487 ***,^*b*^

Diary BG SD^2^	8.9 ± 4.2	8.9 ± 3.8	9.6 ± 4.8	6.4 ± 3.6 **,^a^***,^*b*^

BMI^3^	29.1 ± 5.6	28.2 ± 5.6 *,^*b*^	28.7 ± 3.5	26.5 ± 3.5 **,^a^***,^*b*^

Weight^4^	76.1 ± 16.6	73.8 ± 16.2 *,^*b*^	78.0 ± 10.2	72.2 ± 10.1

Arm skinfold thickness^5^	25.4 ± 10.0	21.0 ± 7.6 **,^*b*^	25.8 ± 9.2	19.9 ± 7.7 ***,^*b*^

Leg skinfold thickness^5^	34.5 ± 13.0	29.7 ± 10.7 **,^*b*^	32.1 ± 12.6	25.1 ± 10.2 ***,^*b*^

Values are expressed as means ± SD. ^1^, years at the beginning of the study. ^2^, Number of subjects who significantly decreased mean pre-meal diary BG. ^3^, Mean pre-meal of diary blood glucose, mg/dL, LBG = lower than 81.8 mg/dL. HBG = higher than 81.8 mg/dL ^4^, Number of subjects who fell into the LBG at end of the study. ^5^, grams/d. *Asterisks *indicate significant differences (Student's *t*-test or Yates test: *, P < 0.05; **, P < 0.01; ***, P < 0.001) *vs*. respective control group values based on "post - pre" measurements (a), or *vs*. baseline values of the same group (*b*). d (vs. control) and e (vs. baseline) refer to P values after intention to treat approach by addition of 11 null values in the HBG treated group.

**Table 2:** Normal- and over-weight groups divided by low and high mean pre-meal diary blood glucose (BG): composition and compliance at baseline and at investigation end.

	NORMAL-WEIGHT							
	Low BG group				High BG group			
	Control		Trained		Control		Trained	
	Baseline	**After 5 mo**.	Baseline	**After 5 mo**.	Baseline	**After 5 mo**.	Baseline	**After 5 mo**.
Number of subjects and gender	5 F + 4 M		16 F +10 M		3 F + 12 M		19 F + 21 M	
Schooling^1^	11.8 ± 3.5		13.3 ± 2.9		10.3 ± 4.7		11.2 ± 3.8	
Age ^1^	27.9 ± 8.2		31.0 ± 8.9		29.3 ± 2.5		29.7 ± 8.9	
Subjects showing BG decrease ^2^	0/9 (0.0%)		3/26 (11.5%)		6/15 (40.0%)		33/40 (82.5%)**,^*a*^	
BG group mean pre-meal^3^	77.3 ± 3.9	79.8 ± 3.7	76.5 ± 3.9	76.7 ± 4.1	90.7 ± 5.2	89.7 ± 6.6	91.4 ± 7.7	80.1 ± 6.6 ***,^a^***,^*b*^
Subjects < 81.8 mg/dL^4^	9	7	26	22	0	1	0	25***,^*a*^
Vegetable intake^5^	228 ± 217	238 ± 226	403 ± 273	504 ± 235	133 ± 151	142 ± 158	247 ± 240	368 ± 246 *,^a^**,^*b*^
Fruit intake^5^	150 ± 122	146 ± 75	246 ± 162	376 ± 346	161 ± 91	143 ± 123	201 ± 157	291 ± 218 *,^a^*,^*b*^

	**OVER-WEIGHT**							
	**Low BG group**				**High BG group**			
	**Control**		**Trained**		**Control**		**Trained**	
	**Baseline**	**After 5 mo**.	**Baseline**	**After 5 mo**.	**Baseline**	**After 5 mo**.	**Baseline**	**After 5 mo**.

Number of subjects and gender	6 F + 2 M		9 F +3 M		7 F + 6 M		14 F + 12 M	
Schooling^1^	11.8 ± 3.0		12.3 ± 2.6		10.6 ± 4.0		11.9 ± 3.3	
Age ^1^	32.8 ± 12.7		35.0 ± 6.7		34.3 ± 15,4		37.5 ± 15.3	
Subjects showing BG decrease ^2^	1/8 (12.5%)		2/12 (16.7%)		2/13 (15.4%)		22/26 (84.6%)***,^*a*^	
BG group mean pre-meal^3^	77.4 ± 3.6	81,8 ± 6.9	77,1 ± 3,1	77,2 ± 4,8	90,9 ± 7,1	93,9 ± 4,8	91,3 ± 6,5	79,6 ± 7.5 ***,^a^***,^*b*^
Subjects < 81.8 mg/dL^4^	8	5	12	10*,^*a*^	0	0	0	19***,^*a*^
Vegetable intake^5^	194 ± 95	373 ± 232	333 ± 169	514 ± 197	278 ± 225	460 ± 284 *,^*b*^	247 ± 160	420 ± 224 ***,^*b*^
Fruit intake^5^	138 ± 121	182 ± 219	214 ± 138	223 ± 124	226 ± 168	167 ± 121	225 ± 116	286 ± 191

Values are expressed as means ± SD. ^1^, years at the beginning of the study. ^2^, Number of subjects who significantly decreased mean pre-meal diary BG. ^3^, Mean pre-meal of diary blood glucose, mg/dL, LBG = lower than 81.8 mg/dL. HBG = higher than 81.8 mg/dL ^4^, Number of subjects who fell into the LBG at end of the study. ^5^, grams/d. *Asterisks *indicate significant differences (Student's *t*-test or Yates test: *, P < 0.05; **, P < 0.01; ***, P < 0.001) *vs*. respective control group values based on "post - pre" measurements (a), or *vs*. baseline values of the same group (*b*).

**Table 3:** Effects of training (IHMP) on diary reports and anthropometry in normal- and over-weight groups divided by low and high mean pre-meal BG.

	NORMAL-WEIGHT							
	Low BG group				High BG group			
	Control		Trained		Control		Trained	
	Baseline	**After 5 mo**.	Baseline	**After 5 mo**.	Baseline	**After 5 mo**.	Baseline	**After 5 mo**.
Energy intake^1^	1794 ± 587	1660 ± 732	1518 ± 586	1357 ± 628	2034 ± 528	1886 ± 417	1852 ± 697	1270 ± 457 **,^a^***,^*b*^
Diary BG SD^2^	8,0 ± 2,4	9,1 ± 1,7	6,3 ± 3,0	5,2 ± 1,8 **,^a^*,^*b*^	8,6 ± 2,2	8,5 ± 2,4	9,1 ± 3,9	6,6 ± 2,5 **,^a^***,^*b*^
BMI^3^	20.3 ± 1,7	21.0 ± 2.8	21.1 ± 1.8	20.7 ± 1.6	20.2 ± 2.3	21.4 ± 2.1	21.8 ± 2.4	20.7 ± 1.9 ***,^a^***,^*b*^
Weight^4^	55.2 ± 7.7	57.0 ± 9.6	57.9 ± 7.8	57.0 ± 7.6	57.5 ± 6.9	60.9 ± 6.4	61.4 ± 10.4	58.9 ± 9.6 ***,^a^***,^*b*^
Arm skinfold thickness^5^	12.9 ± 5.3	14.7 ± 7.7	12.6 ± 6.6	11.3 ± 5.0	11.3 ± 4.3	11.7 ± 4.2	14.1 ± 7.0	11.6 ± 5.7 **,^a^***,^*b*^
Leg skinfold thickness^5^	17.9 ± 8.7	18.6 ± 11.0	17.6 ± 9.3	15.9 ± 7.7	16.0 ± 6.6	15.6 ± 6.5	20.4 ± 10.3	16.2 ± 8.4 **,^a^***,^*b*^
	**OVER-WEIGHT**							
Energy intake^1^	1611 ± 471	1257 ± 629	1618 ± 616	950 ± 448**,^*b*^	1799 ± 701	1343 ± 489 *,^*b*^	1820 ± 570	1123 ± 503 ***,^*b*^
Diary BG SD2	9.1 ± 4.5	8.2 ± 2.6	7.9 ± 2.9	4,8 ± 2.0 **,^*b*^	8.7 ± 4.2	9.4 ± 4.5	10.4 ± 5.4	7.1 ± 4.0 **,^a^**,^*b*^
BMI^3^	29.1 ± 7.9	28.9 ± 7.6	27.9 ± 2.0	26.5 ± 1.9 *,^a^***,^*b*^	29.2 ± 3.9	27.8 ± 4.2 *,^*b*^	29.0 ± 4.1	26.5 ± 4.0,^a^*,***^b^
Weight^4^	74.5 ± 18.3	74.1 ± 17.9	77.0 ± 9.5	73.0 ± 9.1 *,^a^***,^*b*^	77.1 ± 16.2	73.7 ± 15.9 *,^*b*^	78.5 ± 10.6	71.8 ± 10.7 *,^a^***,^*b*^
Arm skinfold thickness^5^	25.3 ± 10.8	23.3 ± 8.7 **,^*b*^	25.9 ± 7.0	21.8 ± 6.4 *,^*b*^	25.5 ± 9.9	19.6 ± 6.8	25.7 ± 10.2	19.09 ± 8.2 ***,^*b*^
Leg skinfold thickness^5^	33.7 ± 13.7	30.3 ± 12.6 **,^*b*^	32.5 ± 12.1	26.6 ± 10.3 ***,^*b*^	34.9 ± 13.1	29.4 ± 9.8**,^*b*^	31.9 ± 13.1	24.4 ± 10.3 ***,^*b*^

^1 ^Kcal/d. ^2^, mg/dL; diary SD refers to BG SD of 21 measurements reported by each of 7d diary. ^3 ^body weight kg/square height meters. ^4 ^Kg ^5 ^mm. *Asterisks *indicate significant differences as in Table 1.

No significant gender difference in baseline mean pre-meal BG concentrations was observed in the control group (females: 83.8 ± 9.2 mg/dL; n = 21; and males: 87,2 ± 7,5 mg/dL; n = 24; Student's t-test for unpaired data: P = 0.24) and in the training group (females: 85.0 ± 8,9 mg/dL; n = 58; and males: 87.2 ± 9.9 mg/dL; n = 46; P = 0.22). The measurements from both genders were thus pooled in each group (Table 1). Baseline mean pre-meal BG for the control subjects (85.6 ± 8.4 mg/dL; n = 45) did not differ from that of the training subjects (86.0 ± 9.4 mg/dL; n = 104; P = 0.80).

### Number of participants

Results were obtained from 149 subjects (79 females and 70 males) randomized into control and training groups (see Methods) and completing the study (Figures 1 and 2).

### Summary of the results

#### OW group

The IHMP was associated with a significant decrease in body weight and BMI in OW subjects compared to controls, after 7-weeks of training and 3 months of application. In the control group BMI significantly decreased from baseline 29.1 ± 5.6 to 28.2 ± 5.6 after 5 months (P = 0.023), however BMI decreased from 28.7 ± 3.5 to 26.5 ± 3.5 in the trained group (pre/post P = 0.0001; comparison in longitudinal differences, P = 0.004). The changes in body weight confirmed BMI results.

MANOVA revealed a significant association between training and both BMI (P = 0.004) and body weight (P = 0.002) variations in the whole OW group. After insertion of the division of this group into LBG and HBG, MANOVA also revealed a significant association between mean BG and both BMI (P = 0.016) and body weight (P = 0.015). An interaction term between division in LBG and HBG groups and training did not reveal any significant change. We analysed by MANOVA training components and their association with BMI and body weight. Mean BG (P = 0.002) resulted as significant factor most involved with the variations in BMI and body weight.

The pre-meal mean BG showed a significant pre-post increase in the whole control group (P = 0.039), in contrast with a significant decrease in the trained group (P = 0.0001 in the pre/post and longitudinal differences between control and trained groups). Diary BG SD remained constant in control group and significantly differed (P = 0.012) from the post-test decrease in the trained group (P = 0.001).

We found no significant difference in the pre/post decrease in energy intake (Student's *t*-test for unpaired data: P = 0.057) and increase in vegetable intake between control and trained groups (P = 0.629) in trained group.

#### NW Group

MANOVA revealed a significant association between training and BMI (P = 0.000), body weight (P = 0.000), arm skinfold thickness (P = 0.001) and leg skinfold thickness (P = 0.008) variations in the NW group. Diary BG SD (P = 0.012) was the factor most significantly associated with variations in arm skinfold thickness.

### Post-hoc analysis - subgroups

Baseline BG mean concentrations were distributed over a wide range. Our results showed substantial weight decreases at study end not only in OW subjects but also in many NW subjects. It appeared that those NW subjects with high baseline BG might account for most of the weight loss shown by NW subjects. It was of interest therefore to use the "cut-off" value (demarcation point) of mean BG concentration that most significantly divided HBG and LBG subgroups in the previous study [16] (81.8 mg/dL) to set apart four subgroups: two subgroups (OW and NW) with low baseline BG (LBG) and two subgroups (OW and NW) with high baseline BG (HBG). Similarly, the BG value of 81.8 mg/dL was used to divide control subjects into OW and NW LBG and HBG control subgroups.

In LBG NW and OW subjects (mean pre-meal BG < 81.8 mg/dL; n = 26 and 12; Table 2) mean pre-meal BG remained constant after training, whereas in HBG NW and OW subjects (mean pre-meal = 81.8 mg/dL; n = 40 and 26; Table 1) mean pre-meal BG significantly decreased. The longitudinal difference was significantly greater than in the control subgroups. In the control subgroups, the BG did not decrease during the study time interval in any of the four subgroups (Table 2).

After 5 months, the number of trained subjects whose mean pre-meal BG fell below 81.8 mg/dL was significantly higher in the two HBG subgroups and in the OW LBG subgroup than in control subjects (Table 2). On the other hand, 22 of 26 NW LBG subjects remained below the BG of 81.8 mg/dL. They did not differ from 7 of 9 control subjects.

The pre/post decreases after training in mean pre-meal BG, diary-BG SD, energy intake, body weight, body mass index (BMI), arm and leg skinfold thickness were all significantly greater in the trained NW HBG group than in the corresponding control subjects (Table 2 and 3). Mean pre-meal BG, diary-BG SD, body weight and BMI also decreased significantly in OW HBG trained subjects compared to controls. Control OW HBG subjects also showed a significantly lower energy intake, body weight and BMI (Table 3), but not mean pre-meal BG (Table 2), at investigation end compared to baseline. The discrepancy prompted us to analyze energy intake, BG and body weight at 7 weeks of investigation. At 7 weeks, daily energy intake was 1082 ± 290 kcal/d and BG 88.0 ± 6.2 mg/dL in control OW HBG subjects. The two values were significantly lower than at investigation end (n = 13, P < 0.02 and 0.01). At 7 weeks, body weight was 72.8 ± 15.3 kg which was significantly lower than at baseline (P = 0.0001) but not than study end.

In the NW LBG group, only the decrease in diary-BG SD was significantly greater than in control subjects in the longitudinal comparison after training. In the OW LBG group, the training was associated with significant pre/post decrease in energy intake, diary BG SD, BMI, body weight, arm and leg skinfold thickness, and the decreases in body weight and BMI were greater than in the OW LBG control group.

Thus the training appeared to decrease weight in OW or HBG subjects while NW LBG subjects maintained normal weight. Moreover, trained OW LBG subjects showed significantly lower energy intake per meal and lower number of meals per day (279 ± 128 kcal per 3.4 ± 0.6; n = 285 meals, P = 0.001) than NW LBG subjects. (367 ± 116 kcal per 3.7 ± 0.7; n = 673 meals) (HBG NW and OW subjects showed no such differences after training).

### Vegetable intake

Vegetable and fruit intake increased significantly in trained NW HBG subjects compared to control subjects. Vegetable intake significantly increased in both trained and control OW HBG subjects without any longitudinal difference between the groups' increases. The longitudinal correlation of vegetable intake vs. energy intake in all trained NW subjects (LBG and HBG together) was significant (ρ = -0.26; P = 0.007; n = 66) and vegetable intake was significant also vs. mean pre-meal BG in all trained OW subjects (ρ = -0.32; P = 0.05; N = 38).

### Well-being, nutrition, and circulation trials

NW subjects showed a non-significant increase in outdoor hours and decrease in diastolic blood pressure compared to control subjects (Table 4). Trained OW subjects showed a significant pre/post decrease in bedtime hours and systolic and diastolic blood pressure. These values decreased but not significantly compared to controls. Trained (NW and OW) LBG groups showed a significant decrease in bedtime hours and in systolic blood pressure, and the longitudinal difference in bedtime hours was significantly greater than in control subjects. The Chi-square analysis for trend toward improvement on the 16 comparisons in systolic and diastolic blood pressure between trained and control subjects (LBG and HBG) was highly significant (P = 0.0001).

**Table 4:** Effects of training on bed time, activity, and blood pressure in HBG groups.

NORMAL-WEIGHT				
	Control		Trained	
	Baseline	**After 5 mo**.	Baseline	**After 5 mo**.
Outdoor and gym hours ^1^	4.8 ± 3.8	3.9 ± 3.4	3.6 ± 2.4	4.1 ± 2.5 *,^*a*^
Bed time ^1^	8.4 ± 0.6	8.4 ± 1.0	8.0 ± 1.1	7.9 ± 1.1
Systolic blood pressure ^2^	115.7 ± 16.1	113.5 ± 12.4	108.2 ± 13.4	103.0 ± 14.1
Diastolic blood pressure ^2^	64.7 ± 12.2	71.0 ± 10.7	66.9 ± 12.4	63.0 ± 11.4**,^*a*^
**OVER-WEIGHT**				
Outdoor and gym hours ^1^	3.6 ± 3.5	3.0 ± 3.0	3.2 ± 3.2	3.7 ± 3.1
Bed time ^1^	7.7 ± 0.6	7.7 ± 0.8	7.8 ± 1.0	7.3 ± 0.9*,^*b*^
Systolic blood pressure ^2^	123.8 ± 18.7	116.2 ± 8.7*,^*b*^	125.4 ± 14.0	112.2 ± 15.3***,^*b*^
Diastolic blood pressure ^2^	73.8 ± 8.7	70.4 ± 11.4	76.3 ± 9.8	68.6 ± 9.5*,^*b*^

^1 ^hours/d; ^2 ^mm Hg; *, **, ***, ^a ^and ^b ^symbols as in Table 2.

### Additional analyses

We contacted 17 of 26 trained HBG OW subjects 9 - 15 years after protocol end. Three subjects decreased body weight from 88.0 ± 6.0 kg to 78.7 ± 7.2 kg after training but showed a mean weight of 96.0 ± 3.5 after 13.3 ± 2.2 years. Fourteen subjects decreased body weight from 78.5 ± 11.2 kg to 73.2 ± 11.4 kg after training. They maintained the IHMP and showed a mean weight of 73.3 ± 13.2 (P = 0.001 Vs. pre-training value) after 10.6 ± 1.8 years. Thus, after 10 years, trained subjects showed a bimodal pattern with most maintaining the IHMP and significant weight loss.

### Adverse events

As in the previous study [16], trained subjects reported few negative effects. Five of 40 NW HBG subjects reported intense hunger at slightly low BG (SLBG, below 60 mg/dL) before five of 840 meals in the diary after training but no fainting. This number of SLBG events was significantly lower than 10/546 meals in 26 OW HBG subjects (P = 0.03). The 10 SLBG events in OW subjects were associated with feelings of faintness in 7 events and transient syncope in 2.

During the first month of training, for 25 of 104 subjects (66 NW and 38 OW) the consumption of the prescribed amounts of fruit and vegetables was followed by diarrhoea in 6 subjects, abdominal pain in 16 and both symptoms in 3. For these subjects, pre-meal BG measured over the previous 6 meals was ≥ 88 mg/dL for one or more meals. When BG so measured over the previous 6 meals was lower than 82 mg/dL, these two symptoms did not follow the prescribed consumption (P < 0.002 for fruit and 0.0001 for vegetables).

## Discussion

### Interpretation of results

#### Synopsis of key findings

A seven-week training program to establish the IHMP led to significant loss of weight in OW subjects and maintenance of weight in NW subjects. Post hoc analysis suggests the IHMP led to loss of weight in subjects who are either OW or who are of NW with HBG. In NW LBG subjects, weight was maintained.

#### Possible mechanisms and explanations

We suggested above (background) that IH may begin an important afferent arm of a physiological regulation mechanism that provides meal-by-meal feedback on energy need thus optimizing energy intake. Subjects who are overweight and those who are normal-weight but have pre-meal HBG forestalled this homeostatic mechanism. Restoring the homeostatic mechanism would explain our finding that the IHMP leads to loss of weight in OW and NW HBG subjects but not in NW LBG subjects

#### Comparison with previous findings

The epigastric sensation of hunger involves motor and secretion activation in the intestine, transient BG drops and activation of anterior cingulate cortex [25-31]. The decrease in mean BG after training implies that before training, OW and NW HBG groups forestalled the activation of this complex function by premature intake and suggests that interoceptive awareness can be improved. With improved interoceptive awareness after training, NW and OW subjects chose to initiate food intake at significantly lower mean pre-meal BG concentrations and lower diary BG SD than before training. After 5 months, only four of 26 trained LBG NW subjects initiated food intake at a mean pre-meal BG of greater than 81.8 mg/dL (and only by few mg/dL). This suggests a pattern of meal intake had been attained that allowed tighter BG control, a pattern that persisted over three months.

Post hoc analysis of pre-meal BG revealed no change in energy-balance habits in subjects who had baseline pre-meal LBG and regression toward LBG after training in those who were OW or had pre-meal HBG. This implies a pre-meal LBG threshold below which hunger is signalled. Previous studies have indicated this threshold occurs at 77.2 ± 4.2 in adults and 75.2 ± 6.9 in infants [11,16,23].

A comparison between children taught to initiate food intake according to either hunger sensation or regular mealtime has been carried out by Birch [32]. In the first group caregivers were instructed to help children become aware of their internal cues of hunger and satiety, and to discuss with the children the relation of such cues to intake regulation. In the second group, the children were obliged to eat on a fixed schedule, being deliberately focused on external cues. The authors found evidence that children who were focused on interoceptive cues later showed an ability to adjust their intake to the actual energy content of ingested foods whereas the children who were focused on exteroceptive cues showed no such ability.

### Limitations of the study

#### Modified Intention to treat analysis

The most important limitation of this study is the high number of subjects (n = 32) who did not complete the study (dropouts) after the first two months. Twenty six of these 32 subjects were trained and confirmed that they had experienced improvements but left citing their busy schedule or no felt need for further instructions. In intention to treat analysis those subjects who do not follow a study protocol are included in the final analysis. We included all subjects who enrolled for the study and for whom we have end-point data, however a number of subjects were lost to follow-up so our findings represent a modified intention to treat analysis. The likely effect of those subjects who dropped out was assessed by sensitivity analysis.

#### Sensitivity analysis

We have data on all 32 dropouts (26 trained and 6 control subjects) from the 7 week post-training visit (Figure 1 and 2). The data showed agreement with the group that fulfilled the protocol with respect to mean BG, energy intake, BMI and body weight in 7 LBG NW subjects and 6 HBG NW subjects. Three LBG OW subjects together with 10 HBG OW subjects showed significant decrease in mean BG, energy intake and body weight. The six control dropouts showed no change in these assessments. From these data we conclude that the dropout subjects are unlikely to represent a significantly different population in respect to the endpoint measures of this study and that the absence of final data from these subjects is unlikely to have significantly affected the result overall.

#### Training period and 7-day diaries

Subjects were asked to identify IH and base their decision to eat on its presence. During training, BG concentration was used as an objective validation of the subjective experience immediately after the experience was identified. The intervention in this study is IH and the outcome is weight. BG is an intermediate variable and it must be acknowledged that in completing their diaries during the final week, trained subjects also measured BG concentration. However, before measurement they estimated BG on the basis of IH, an estimation they were able to perform accurately [11]. Glycated haemoglobin reflects the average BG over a 4 month period and the lowered glycated haemoglobin in the previous study [16] and significant weight loss observed in this study are unlikely to have occurred in the final week. These data suggest that awareness of IH indeed preceded BG measurement, and was not significantly affected by it.

#### Generalizability

Our findings are upon subjects who attended a gastroenterology clinic over a 5 month period. Further investigation will be necessary to evaluate the effect of the IHMP in other populations and what "reminder" training might be necessary to ensure compliance with the IHMP and maintenance of body weight maintenance over years [21,22].

### Clinical and research implications

#### Advantages over conventional dieting

##### Restraint approach

Control subjects were encouraged to lose weight and can be considered to represent a conventional restraint approach to dieting. Although control OW HBG subjects significantly lost weight in the first two months they significantly increased their energy intake and BG during the last three months of the study and lost no further weight. This is consistent with a "restrained" eating pattern. Control OW LBG subjects showed a mean pre-meal BG just at 81.8 mg/dL at the end of the study indicating that without training, their meals remained partly conditioned, thus explaining firstly, their overweight status, and secondly, their failure to lose weight. Thus the findings in the two control OW subgroups (LBG and HBG) are consistent with the fact that restraint-type dieting tends to give short term results that are not sustained.

Weight cycling is a well-described phenomenon [33]. In the first phase of the cycle intake is conditioned or non-homeostatic. This leads to positive energy balance and weight increase. In the second phase OW subjects restrain their eating to lose weight. Most likely, the OW LBG subgroup was in this second phase at baseline. In the post-absorptive state, OW subjects have been shown to mobilise greater amounts of energy from reserve tissues to blood compared to NW subjects [34]. By attending to preprandial arousal of IH, trained OW LBG subjects had to adjust meal energy intake downwards sufficiently to take into account the increased availability of energy owing to postabsorptive energy release, hence their lower energy intake (about 300 kcal per day) compared to trained LBG NW subjects. During established IHMP, OW subjects reported that, provided meals were not delayed, their hunger was of no greater intensity nor more prolonged than NW subjects. Moreover, despite significantly higher body weight and lower energy intake than NW LBG subjects, trained OW LBG subjects showed the same mean preprandial BG as trained NW subjects (Table 2, 3). These findings have at least three important clinical and research implications:

1. Trained OW subjects do not need to endure more prolonged or more intense hunger than NW subjects in order to lose weight.

2. The IHMP allows loss of weight without compromising energy availability for day-to-day energy need. The input of fatty acids from fat tissues to blood is limited in the overweight. Diets with lower mean content than 900 kcal a day may yield insufficient energy for body functions. That preprandial BG in the OW LBG group was the same as the NW LBG group indicates that in the OW LBG group a sufficiently high BG concentration was maintained for immediate energy needs. SD of diary BG in trained OW groups significantly decreased and regressed to that of NW groups further suggesting that under the IHMP OW groups adapted energy intake to metabolic need. In the absence of energy deprivation, less cycling of intake among trained OW groups would be expected.

3. An important subgroup exists (NW HBG) who appear NW by BMI criteria but who may nevertheless be at risk of weight related complications since they lose weight and decrease BG to a concentration comparable to the LBG group when trained in unconditioned eating.

##### Food composition approach (increased vegetables)

After 5 months, no significant difference was found in vegetable intake between control and trained subjects. At the end of the study controls did not attain significantly lower BG or body weight than the trained group although they had been encouraged to lose weight. This implies that high vegetable intake alone is insufficient in preventing conditioned meals and lowering high BG.

#### Sleep and the IHMP

Restriction of bedtime (4 hours per day per 6 days vs.12 hours per day per 6 days) has been associated with cardiovascular risk factors including impaired insulin sensitivity [35]. Our IHMP-trained subjects showed a small but significant decrease in sleep hours compared to controls yet in a previous study the IHMP was associated with improved insulin sensitivity [16]. We suggest therefore that the observed decrease in sleep hours do not represent sleep debt but rather a physiological lowered sleep requirement associated with homeostatic eating. The mechanism by which this might occur is not yet clear.

#### Advantages of immediate feedback

Subjects following the IHMP receive meal-by-meal subjective feedback from physiological signals. These signals map closely to BG and allow subjects to eat in an unconditioned manner without self-imposed restraint or the necessity to seek any particular goal weight. The resulting improved energy balance leads to loss of weight. "Normal weight" is an artificial construct based on population statistics and may not apply to a given individual.

Recommendations of goal weight may be unhelpful for some subjects to whom the goal may seem arbitrary and daunting especially if it is to be achieved by dietary restraint. The IHMP obviates the need for pursuit of a statistical norm and allows each individual to find his or her physiological norm.

This approach could thus prove useful in the clinical setting since it removes major obstacles to weight loss - the need for restraint, the need for dietary change, and the need to attain an arbitrary weight goal.

#### General interpretation

The IHMP is an easy learned and reliable method to promote and maintain unconditioned eating. Our findings suggest that patients can maintain this eating pattern without further training for months, that it leads to improved insulin sensitivity [16] and that it promotes weight loss in OW subjects. The IHMP could therefore be an important tool in the clinical management of overweight and obese patients and could have implications for health policy in the prevention of a wide range of metabolic and vascular disorders.

## Conclusion

A three-times- daily meal pattern (IHMP) was associated with LBG and sustained regression of overweight. The method was more effective than restraint-type dieting in a 5 month trial. IH, validated by BG, may represent the recovery of a vital afferent arm of the body's homeostatic energy regulation system allowing sustained self-regulation of energy intake. Post hoc division of NW and OW subjects into subgroups with mean pre-meal BG either lower or higher than 81.8 mg/dL suggests body weight maintenance in NW subgroup with low mean BG and decrease in those who were either OW or HBG NW.

The findings of this study and those of the accompanying study [16] suggest that the current epidemic of insulin resistance and overweight may have its origin in the non-cognizance of hunger - the physiological signals of energy insufficiency to body cells. This may owe to forestalling such signals in early life and subsequent reinforcement of this behaviour pattern. By restoring and validating hunger awareness, the IHMP could help in the prevention and treatment of diabetes and obesity and a range of associated disorders and thus lessen the high economic burden of health services in industrialized societies.

## List of abbreviations

BG: Blood glucose; LBG: Low mean pre-meal blood glucose concentration (below 81.8 mg/dL); HBG: High mean pre-meal blood glucose concentration (over 81.8 mg/dL); BG estimation: During training: writing the expected BG value just prior to measuring the blood sample by glucometer. After training and validation: subjectively evaluating own current BG value without measurement; Mean BG: Mean pre-meal blood glucose as reported by seven day diary; Diary-BG SD: Mean pre-meal blood glucose standard deviation as reported by seven day diary. Seven day diaries were completed at baseline, 7 weeks and 5 months; NW: Normal weight (BMI below 25.0); OW: Overweight (BMI over 25.0); BMI: Body mass index; IH: Initial hunger; IHMP: Initial hunger meal pattern.

## Competing interests

The authors declare that they have no competing interests.

## Authors' contributions

MC conceived and performed the investigation. DLS contributed to interpretation of data and drafting the paper. MS participated in the design of the study and performed the statistical analyses. All authors read and approved the final manuscript.
